# A protocol for the development and piloting of quality measures to support the Healthier You: The NHS Diabetes Prevention Programme

**DOI:** 10.3399/bjgpopen17X101205

**Published:** 2017-11-01

**Authors:** Farina Kokab, Rachel Foskett-Tharby, Nick Hex, Paramjit Gill

**Affiliations:** 1 Research Associate, Institute of Applied Health Research, University of Birmingham, Birmingham, UK; 2 Institute of Applied Health Research, The University of Birmingham, Birmingham, UK; 3 York Health Economics Consortium, University of York, York, UK; 4 Institute of Applied Health Research, The University of Birmingham, Birmingham, UK

**Keywords:** Type 2 Diabetes, quality measures, pilot, protocol, non-diabetic hyperglycaemia, national diabetes prevention programme

## Abstract

**Background:**

The increasing prevalence of type 2 diabetes in the UK creates an additional, potentially preventable burden on health care and service providers. The Healthier You: NHS Diabetes Prevention Programme aims to reduce the incidence of type 2 diabetes through the identification of people at risk and the provision of intensive lifestyle change support. The provision of this care can be monitored through quality measurement at both the general practice and specialist service level.

**Aim:**

To develop quality measures through piloting to assess the validity, credibility, acceptability, reliability, and feasibility of any proposed measures.

**Design & setting:**

The non-experimental mixed design piloting study consists of consensus testing and exploratory research with GPs, commissioners, and patients from Herefordshire, England.

**Method:**

A mixed-method approach will be used to develop and validate measures for diabetes prevention care and evaluate their performance over a 6-month pilot period consisting of consensus testing using a modified RAND approach with GPs and commissioners; four focus groups with 8–10 participants discussing experiences of non-diabetic hyperglycaemia (NDH), perceived ability to access care and prevent diabetes, and views on potential quality measures; and piloting final measures with at least five general practices for baseline and 6-month data.

**Results:**

The findings will inform the implementation of the diabetes prevention quality measures on a national scale while addressing any issue with validity, credibility, feasibility, and cost-effectiveness.

**Conclusion:**

Healthcare professionals and patients have the opportunity to evaluate the reliability, acceptability, and validity of measures.

## How this fits in

Relevant National Institute for Health and Social Care Excellence (NICE) guidelines and wider literature have identified potential measures to prevent patients identified with NDH progressing to diabetes or to help manage their condition. The application of these measures will be piloted with clinicians, to identify their suitability. 

## Introduction

The growing incidence and management of type 2 diabetes presents a significant cost to the NHS in England.^1^ Type 2 diabetes is a preventable chronic disease, and lifestyle change can help reduce risk.^2^

Patients with NDH have raised levels of Hb1Ac or fasting glucose but these are not high enough to be diagnosed with type 2 diabetes.^3^ Intensive lifestyle modification programmes or interventions can target people with NDH in order to delay or prevent the onset of type 2 diabetes and associated morbidity.^4^ The efficacy of lifestyle interventions requires further investigation, alongside evaluation of diabetes incidence and risk reduction in health outcomes.^5–8^

As part of the *Five Year Forward View*, the NHS and Public Health England has announced the development and implementation of a diabetes prevention programme to support people managing their own health.^9^ As part of this programme, interest has been expressed in developing quality measures suitable for use at the general practice and clinical commissioning group (CCG) level for those at risk of developing diabetes. The National Collaborating Centre for Indicator Development (NCCID) works on behalf of the NICE to develop and evaluate quality measures of primary care. Within primary care they have also been used to incentivise certain aspects of care through the Quality and Outcomes Framework (QOF).^10^

Evaluation has been part of the NCCID protocol since 2009 and is a recommended step by the National Committee for Quality Assurance.^11^ Piloting measures can help identify any concerns prior to use in assessment frameworks, thus giving time to adapt indicator wording or measurement protocols to address issues such as tunnel vision, gaming, misinterpretation, and measure fixation.^12^

### Quality measures

The desirable attributes of quality measures are validity, reliability, and acceptability to those being assessed and those making the assessment, as well as feasibility, and cost-effectiveness^11,13–17^ (Box 1).

**Box 1.** Desirable attributes of quality measures

Attribute	Definition	Importance
Content and construct validity	The ability of the measure to accurately capture the quality of care being delivered The measure being able to cover important aspects of care that contribute to perceptions of care quality The extent to which performance on a measure correlates (positively or negatively) with other aspects of care that theories suggest it should correlate with	To ensure that the quality measures reflect the aspects of care interest to members of the public, providers, and/or regulators
Reliability	The accuracy with which a measure can produce similar results under different conditions (including people applying the measure, and the situation in which it is applied)	To ensure that differences in performance between providers and over time are a true reflection of differences in care
Feasibility	The ability to accurately measure the quality indicator with ease and efficiency	To ensure that the information required in order to calculate achievement against the quality measure can be collected easily and efficiently. If measures require too much time, money or effort then they may not be suitable for use

Measures can be developed to evaluate structure (features of an organisation), process (related activities), and outcome (change in health status),^11,13^ as appropriate.

### Aims and objectives

The aim of this unique work is to develop and evaluate a set of quality measures for the identification and management of people with NDH that are suitable for use in England at the general practice and CCG levels, as well as research questions that are relevant to clinical practice. The findings from the focus groups will provide clinicians with insight into patients' perspectives on and experiences of communication and management of NDH. The specific objectives are:

1. to develop a comprehensive set of quality measures based on relevant NICE guidance and the evidence of reviews underpinning NHS England’s Diabetes Prevention Programme;
2. to undertake preliminary evaluation of the validity and feasibility of these measures through the use of a RAND consensus methodology;
3. to explore patients’ experiences of being identified with NDH and views on quality outcome measures;
4. to develop and test data extraction specifications to support these measures at general practices including IT system compatibility; and
5. to evaluate the resulting measures in a 6-month pilot.

## Method

Relevant NICE guidelines will be identified and used to underpin measure development alongside evidence reviews supporting the development of the Diabetes Prevention Programme.^18,19^ Recommendations from these will be developed into quality measure statements initially with an 'IF, THEN, BECAUSE' approach to ensure clarity in terms of population of interest; the care activity to be undertaken; the circumstances under which this activity should be performed, for example duration; and the rationale (Box 2).^19^

**Box 2.** Quality measurement statement

IF, THEN, BECAUSE statement	IF a patient has been identified as having NDH (HbA1c 6.0–6.4%/42–47 mmol/mol) THEN they should be offered a blood test at least once a year BECAUSE they may develop type 2 diabetes
Quality measure statement	The percentage of patients with NDH who have had an HbA1c or fasting plasma glucose in the preceding 12 months
Denominator	Patients with NDH
Numerator	Patients identified with NDH for whom there is a record of either an HbA1c or fasting plasma glucose being measured in the preceding 12 months

A comprehensive set of quality measures will be developed to address the multifaceted nature of quality and the different organisational structures involved in the provision of this care, incorporating statements about structure, process, or outcomes to generate review criteria and standards that help measure quality.^20^ Quality assessment can include processes (activities undertaken by the provider), outcomes (health or events following care), and risk adjustment (factors outside of the health system [patient demographics, illnesses, treatment, and organisation]).^21^

### RAND consensus methodology

The RAND appropriateness method uses a group judgment process to systematically and quantitatively incorporate expert opinion with scientific evidence, whereby panellists rate, discuss, and re-rate quality measures.^20^ Benefits include the opportunity to discuss issues and maintain all potential information, as no indicators are dropped between meetings, as well as the use of scientific literature. The RAND panel will be a convenience sample of GPs. Recruitment to the RAND panels will be open to GPs from diverse backgrounds; for example, those with recent qualifications versus long-standing registered GPs, salaried versus partnered GPs. However, panels can be small, patients are rarely involved, and there is potential for dominant personalities to carry more influence.^20^

In a systematic review by Kotter *et al,* a consensus method was used in all 35 included studies, 15 of which used a modified RAND method to limit stakeholder influence and incorporate expert opinion.^22^ The use of RAND in UK quality measurement has been outlined in research for conditions such as angina and non-insulin dependent diabetes in general practice settings.^23^

The research panels will be undertaken with GPs and CCG commissioners which could result in missing out patient experiences, but there is a risk in combining patient and professional views as patients may be dominated by professionals.^24^

The clarity, validity, and feasibility of identified measures will be evaluated using a modified RAND consensus methodology with groups of GPs and CCG level commissioners within Herefordshire CCG. A modified RAND methodology offers a unique systematic approach to synthesising the evidence base underpinning the quality measures, using existing literature as well as professional opinions.^25^ It has been widely used to develop quality measures in the UK and internationally.^26^

**Figure 1.**
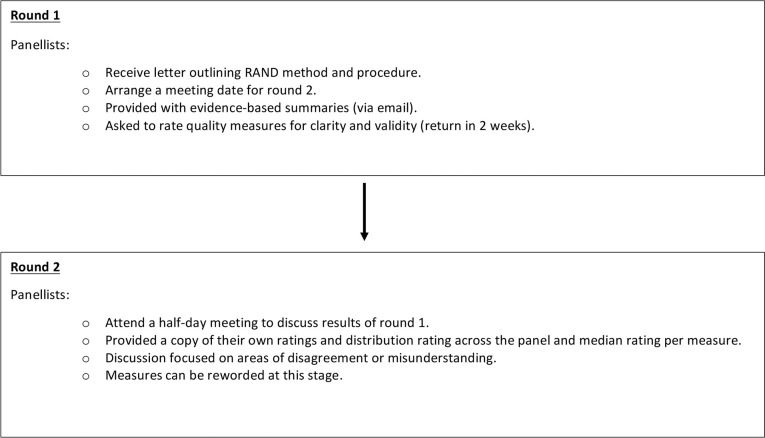
RAND consensus methodology process for panellists.

Each RAND panel will consist of two rounds of quality measure ranking (Figure 1).

### Explore patient views

Qualitative research with patients lends itself to the enhancement of the validity and clarity of measures, not only for providers but also patients who face the consequences of being identified with the condition and of being part of the healthcare system.

There is a diverse range of behaviour change theories that can help develop an understanding of constructs regarding people’s behaviour.^27^ The COM-B model provides three factors — capability, opportunity and motivation — to develop behavioural targets as a basis for intervention design.^28^ The model encompasses a variety of psychosocial factors that can determine initiation and maintenance of behavioural change.^29^ The model has been applied successfully in a number of contexts to understand behaviour change.^28^ By investigating patient views and behaviours in relation to health management, researchers can identify potential barriers to or facilitators for changing behaviour once an identification of chronic disease risk has been made.

Although patient involvement in measure development has been carried out, there is a limited number of works in this field, especially regarding details of involvement and representativeness.^22^

Patient views will be explored through a minimum of four focus groups, each consisting of 8–10 participants. Braun and Clarke suggest 3–6 focus groups can provide sufficient data for a medium sized project.^28^ Data collection will be an iterative process where focus group recordings will be transcribed and analysed to monitor data saturation and provide additional topics for subsequent focus groups.^30^ Incorporating patient views will further strengthen understanding of quality measure suitability. Furthermore, the use of the COM-B model of behaviour change to guide data collection, analysis, and interpretation will provide findings that can be understood from different healthcare perspectives, for example, for health interventions considering patient views on preventative care.^29^

Patients will be identified through direct recruitment from GP practices or through local support networks as eligible for the focus groups if they:

- have been recorded as having NDH or their latest HbA1c was between 42–47 mmol/mol in the last 6 months (Table 1); and
- are aged ≥18 years

**Table 1.** Diagnostic threshold table HbA1c (fasting glucose)

Risk	%	mmol/mol
Normal	<6.0	<42
Pre-diabetes/NDH	6.0–6.4	42–47
Diabetes	≥6.5	≥48
Exclusion: Patients will be excluded if their HbA1c is within the pre-diabetes/NDH range (6.0–6.4) but they are known to have diabetes or impaired glucose tolerance as a result of pregnancy. The resulting list will be reviewed by a GP in the practice to identify and remove patients whom it would be inappropriate to contact, for example,. those at end of life.		

The practice will be asked to send patients information sheets about the study and an invitation to participate. No personal information will leave the practice/CCG. Patients will be contacted if they are at risk and inclusion will not be limited to patients taking part in the diabetes prevention programme.

The focus group will begin with an initial discussion of people’s experiences of diabetes prevention care and then each potential measure will be presented to the group with the reason for its development.

The topic guide will be used to gather data on participant's views and experiences, and is structured based on the three integral components of the COM-B model — namely, capability (both psychological and physical), motivation, and opportunity (physical and environmental)^29^ (Box 3).

**Box 3.** Questions and probes from the topic guide

**Example of an opening question** What do you think good care is? How would you describe the quality of the care that you receive?
**Example of a question regarding patient’s emotions** What does it feel like to have pre-diabetes as a label in your records? Were you expecting this? Why?
	**Discussion on quality indicators** What do you think about repeating blood tests? What do you feel about this?

Participants' experience of risk assessment can parallel the experience of illness and associated social implications.^31^ Participants will therefore be encouraged to make suggestions not only to improve clarity and acceptability of the potential measures for diabetes prevention, but also to shed light on their experiences and perception of having an elevated blood sugar level and their capability, motivation, and opportunities in relation to it. Analysis of participants’ comments will be an iterative process and run concurrently through data collection.

### Quality measure piloting and evaluation

A convenience sample of five GP practices will be recruited in NHS Herefordshire CCG to a maximum of 24 (whole health economy).

Practices which agree to take part will be expected to:

1. Work with the quality measure for the 6 months of the pilot.
2. Complete baseline and end of pilot computerised data extraction of quality measure achievement;
  - electronic data will be extracted for participating practices using EMIS web queries at the beginning and end of the pilot period in order to assess how performance against the measure changed over time. Demographic data will be extracted to explore potential differences as a result of age, sex, and ethnicity.
3. Complete a workload diary for 1 month of the pilot;
  - these consist of completing a spreadsheet that monitors how much time was spent on each quality measure.
4. Complete an end of pilot interview.

The lead GP at each practice and any other staff, including diabetic nurses who were actively involved in piloting, will be interviewed at the end of the pilot.

All interviews will be audiorecorded and transcribed verbatim. Data will be analysed thematically.^32^

### Cost-effectiveness analysis

For any QOF indicators developed from the agreed measures, their cost-effectiveness will be determined using a net (monetised) benefit approach.^33^ In summary, the method applies the following calculation to an indicator:

**Net benefit = (monetised health benefit – delivery cost) – QOF payment.**

QOF payments are an incentive paid to GP practices when certain quality thresholds relating to the indicators are achieved.

The costs and benefits for each indicator are estimated through a rapid review of the available economic evidence. Evidence of delivery costs and benefits, expressed as quality-adjusted life years (QALYs), is sought. The opinions of those involved in delivering the indicators at pilot GP sites are also sought.

The costs associated with an indicator include service delivery costs; for example, the costs of additional GP or nurse consultations to monitor a patient’s health status over a period of time, the initial cost of implementing the intervention, and, if relevant, secondary care service usage. Unit costs will either be extracted from the literature review or derived from published sources such as NHS Reference Costs.

The benefits refer to health benefits which might be gained by a patient as a result of the introduction of the indicator. Effectiveness will be derived from the literature in the form of utility values, which can be converted to QALYs. The monetised health benefit of the indicator refers to the value of the health improvements associated with achieving the predicted benefits of the indicator. These benefits are presented in terms of the changes in QALYs as a result of introducing the new indicator compared to standard practice without the new indicator.

Sensitivity analysis is performed through simple scenario analysis rather than probabilistic sensitivity analysis.

### Practical issues

The focus groups will be conducted with two members of the research team and all focus group interviews will be analysed and verified among the team to limit research subjectivity or bias of one researcher.^34^

Participants are free to withdraw from the research at any time without giving any reason. There is no intervention, manipulation or deception taking place.

## Discussion

### Summary

The study undertakes the piloting of quality measure validity, credibility, and feasibility as part of the diabetes prevention programme.

### Strengths and limitations

The strengths of this study include: a practical design; patient evaluation, which is rarely carried out and will contribute to a wider understanding of identification of NDH and consequences (if any) on quality of life; a theoretical framework to inform data collection in the focus group; exploration of patient experiences to provide insight into identification of NDH and measure appropriateness; and piloting, which will allow the evaluation of quality measures before they are implemented as part of a longitudinal national project. Insight into patient experiences and perspectives on quality measures will help care providers and health services to understand the effects of identification and its consequences for patients’ lifestyle, engagement with care services, and overall wellbeing.

Nonetheless, there are challenges with recruiting and retaining participants across a multi-stage piloting process. GP practices within the overall Herefordshire health economy will be approached and potentially recruited to participate in the study. The identification by the GP of patients meeting the inclusion criteria and the inviting of participants to take part in the research may be limited to participants who are actively aware of their risk and not apprehensive about discussing it.

Managing practices to collect baseline and final measurements from patients may create potential difficulties. This will require a considerable amount of resources to meet the recruitment targets and involve collaboration with NHS Herefordshire. Any issues with non-responsive patients will be discussed with the practice manager.

The RAND methodology is also limited in its ability to incorporate patient views, but this study aims to address this by carrying out a number of focus group sessions to gather data on patients' experiences of being identified as having NDH and their views on quality measures. Although RAND panels may be limited in group size and the piloting focus is restricted to one geographical area (Hereford) that is not representative of England, the research will not only gauge expert opinion, but will also incorporate patient views on these measures and any implications which identification of pre-diabetes has for their emotional wellbeing.

Herefordshire CCG is a first wave implementation site of the diabetes prevention programme. The population provides the opportunity to sample individuals from a lower rural socioeconomic status. However, Herefordshire is a relatively homogenous area in terms of ethnicity and this could limit generalisability of the focus groups. Focus groups should be replicated in more diverse areas.

The focus of the current pilot is not on outcome-driven quality measures in diabetes prevention care, as they are not normally rewarded. The findings may suggest alternative measures need to be implemented to support process and risk adjustment factors.

The pilot will contribute to the evidence base about quality measure piloting and implementation, but also inform the Diabetes Prevention Programme about barriers and facilitators to the management of care for people with NDH.

## References

[1] England NHS (2014). Action for diabetes.

[2] Bagust A, Hopkinson PK, Maslove L (2002). The projected health care burden of Type 2 diabetes in the UK from 2000 to 2016. Diabet Med.

[3] Saudek CD, Herman WH, Sacks DB (2008). New look at screening and diagnosing diabetes mellitus. J Clin Endocrinol Metab.

[4] Ashra NB, Spong R, Carter P (2015). A systematic review and meta-analysis assessing effectiveness of pragmatic lifestyle interventions for the prevention of type 2 diabetes mellitus in routine practice. https://www.gov.uk/government/uploads/system/uploads/attachment_data/file/456147/PHE_Evidence_Review_of_diabetes_prevention_programmes-_FINAL.pdf.

[5] Dunkley AJ, Bodicoat DH, Greaves CJ (2014). Diabetes prevention in the real world: effectiveness of pragmatic lifestyle interventions for the prevention of type 2 diabetes and of the impact of adherence to guideline recommendations: a systematic review and meta-analysis. Diabetes Care.

[6] Knowler WC, Fowler SE, Diabetes Prevention Program Research Group (2009). 10-year follow-up of diabetes incidence and weight loss in the Diabetes Prevention Program Outcomes Study. Lancet.

[7] Orozco LJ, Buchleitner AM, Gimenez-Perez G (2008). Exercise or exercise and diet for preventing type 2 diabetes mellitus. Cochrane Database of Syst Rev.

[8] Aguiar EJ, Morgan PJ, Collins CE (2014). Efficacy of interventions that include diet, aerobic and resistance training components for type 2 diabetes prevention: a systematic review with meta-analysis. Int J Behav Nut Phys Act.

[9] NHS England (2014). Five year forward view: time to deliver. https://www.england.nhs.uk/wp-content/uploads/2014/10/5yfv-web.pdf.

[10] Gill P, Foskett-Tharby R, Hex N (2015). Pay-for-performance and primary care physicians: lessons from the U.K Quality and Outcomes Framework for local incentive schemes. JRSM.

[11] Campbell SM, Kontopantelis E, Hannon K (2011). Framework and indicator testing protocol for developing and piloting quality indicators for the UK quality and outcomes framework. BMC Fam Pract.

[12] Lester HE, Hannon KL, Campbell SM (2011). Identifying unintended consequences of quality indicators: a qualitative study. BMJ Qual Saf.

[13] Rushforth B, Stokes T, Andrews E (2015). Developing 'high impact' guideline-based quality indicators for UK primary care: a multi-stage consensus process. BMC Fam Pract.

[14] McShane M, Mitchell E (2015). Person centred coordinated care: where does the QOF point us?. BMJ.

[15] Streiner D, And Norman G (2009). Health measurement scales: a practical guide to their development and use.

[16] Young GJ, White B, Burgess JF (2005). Conceptual issues in the design and implementation of pay-for-quality programs. Am J Med Qual.

[17] Doran T, Fullwood C, Gravelle H (2006). Pay-for-performance programs in family practices in the United Kingdom. N Engl J Med.

[18] Orchard TJ, Temprosa M, Diabetes Prevention Program Outcomes Study Research Group (2013). Long-term effects of the Diabetes Prevention Program interventions on cardiovascular risk factors: a report from the DPP Outcomes Study. Diabet Med.

[19] MacLean CH, Saag KG, Solomon DH (2004). Measuring quality in arthritis care: methods for developing the Arthritis Foundation's quality indicator set. Arthritis Care Res.

[20] Campbell SM, Braspenning J, Hutchinson A (2002). Research methods used in developing and applying quality indicators in primary care. Qual Saf Health Care.

[21] Mainz J (2003). Defining and classifying clinical indicators for quality improvement. Int J Qual Health Care.

[22] Kötter T, Blozik E, Scherer M (2012). Methods for the guideline-based development of quality indicators--a systematic review. Implement Sci.

[23] Steel N, Melzer D, Shekelle PG (2004). Developing quality indicators for older adults: transfer from the USA to the UK is feasible. Qual Saf Health Care.

[24] Uphoff EP, Wennekes L, Punt CJ (2012). Development of generic quality indicators for patient-centered cancer care by using a RAND modified Delphi method. Cancer Nurs.

[25] Rubin HR, Pronovost P, Diette GB (2001). From a process of care to a measure: the development and testing of a quality indicator. Int J Qual Health Care.

[26] Bell BG, Spencer R, Avery AJ (2014). Tools for measuring patient safety in primary care settings using the RAND/UCLA appropriateness method. BMC Fam Pract.

[27] Barker F, Atkins L, de Lusignan S (2016). Applying the COM-B behaviour model and behaviour change wheel to develop an intervention to improve hearing-aid use in adult auditory rehabilitation. Intl J Audiol.

[28] Braun V, Clarke V (2013). Successful qualitative research: a practical guide for beginners.

[29] Michie S, van Stralen MM, West R (2011). The behaviour change wheel: a new method for characterising and designing behaviour change interventions. Implement Sci.

[30] Achterberg CL, Arendt SW, Monsen ER,, Van Horn L, eds. (2007). *The philosophy role, and method of qualitative inquiry in research.*. Research: successful approaches.

[31] Gillespie C (2015). The risk experience: the social effects of health screening and the emergence of a proto-illness. Sociol Health Illn.

[32] Braun V, Clarke V (2006). Using thematic analysis in psychology. Qual Res Psychol.

[33] Qureshi N, Weng S, Hex N (2016). The role of cost-effectiveness analysis in the development of indicators to support incentive-based behaviour in primary care in England. J Health Serv Res Policy.

[34] Pope C, Ziebland S, Mays N (2000). Analysing qualitative data. BMJ.

